# Oyster Hydrolysates Attenuate Muscle Atrophy via Regulating Protein Turnover and Mitochondria Biogenesis in C2C12 Cell and Immobilized Mice

**DOI:** 10.3390/nu13124385

**Published:** 2021-12-08

**Authors:** So-Hyun Jeon, Se-Young Choung

**Affiliations:** 1 Department of Biomedical and Pharmaceutical Sciences, Graduate School, Kyung Hee University, 26, Kyungheedae-ro, Dongdaemun-gu, Seoul 02447, Korea; yrs02223@daum.net; 2 Department of Preventive Pharmacy and Toxicology, College of Pharmacy, Kyung Hee University, 26, Kyungheedae-ro, Dongdaemun-gu, Seoul 02447, Korea

**Keywords:** oyster hydrolysates, muscle atrophy, C2C12 cells, skeletal muscle, protein turnover, mitochondrial biogenesis

## Abstract

Sarcopenia, also known as skeletal muscle atrophy, is characterized by significant loss of muscle mass and strength. Oyster (Crassostrea gigas) hydrolysates have anti-cancer, antioxidant, and anti-inflammation properties. However, the anti-sarcopenic effect of oyster hydrolysates remains uninvestigated. Therefore, we prepared two different oyster hydrolysates, namely TGPN and PNY. This study aimed to determine the anti-muscle atrophy efficacy and molecular mechanisms of TGPN and PNY on both C2C12 cell lines and mice. In vitro, the TGPN and PNY recovered the dexamethasone-induced reduction in the myotube diameters. In vivo, TGPN and PNY administration not only improved grip strength and exercise endurance, but also attenuated the loss of muscle mass and muscle fiber cross-sectional area. Mechanistically, TGPN and PNY increased the expression of protein synthesis-related protein levels via phosphoinositide 3-kinase (PI3K)/protein kinase B (Akt)/mammalian target of the rapamycin pathway, and reduced the expression of protein degradation-related protein levels via the PI3K/Akt/forkhead box O pathway. Also, TGPN and PNY stimulated NAD-dependent deacetylase sirtuin-1(SIRT1), peroxisome proliferator-activated receptor gamma coactivator-1 alpha (PGC-1α), nuclear respiratory factor 1,2, mitochondrial transcription factor A, along with mitochondrial DNA content via SIRT1/PGC-1α signaling. These findings suggest oyster hydrolysates could be used as a valuable natural material that inhibits skeletal muscle atrophy via regulating protein turnover and mitochondrial biogenesis.

## 1. Introduction

The skeletal muscles, the largest metabolic tissue in the body, significantly contribute to the body’s overall energy balance, and the mitochondria’s oxidative function [1]. Sarcopenia, also known as skeletal muscle atrophy, is an age-associated condition characterized by the significant loss of muscle mass and function [2,3], and muscle fiber cross-sectional area (CSA), thereby restricting physical activities of affected patients, and reducing their quality of life [4]. Disabilities associated with muscle atrophy have also been reported, due to the decrease in protein content, fiber diameter, fatigue resistance, and force production [5,6,7,8,9]. The worldwide prevalence of sarcopenia in individuals over the age of 60 is approximately 5–13% [10], whereas in those over the age of 50, the annual loss of muscle mass ranges between 1–2%, with a muscle strength decrease of approximately 1.5% per year between the ages of 50 and 60 years, and 3% beyond the age of 60 [11]. As life expectancy has increased, sarcopenia has attracted attention since it reduces the quality of life of those impacted.

Skeletal muscle mass mainly depends on muscle fiber size and protein turnover. Skeletal muscle fibers are classified into fiber types, namely slow-twitch (type I) and fast-twitch (type II), distinguished from myosin heavy chain (MyHC) and ATPase isoforms [12,13,14]. Slow-twitch muscle fibers contract slowly, have a high content of mitochondria, and primarily rely on oxidative metabolism as an energy source. In contrast, fast-twitch muscle fibers rapidly contract, have a low content of mitochondria [15], and mainly depend on glycolysis metabolism as a source of energy. The soleus muscle is predominantly slow-twitch muscle fibers, whereas the quadriceps and gastrocnemius muscles are predominantly fast-twitch muscle fibers [16,17,18]. During muscle inactivity, atrophic slow-twitch fibers are reduced compared to fast-twitch fibers [19]. Simultaneously, the fiber type shifts from slow- to fast-twitching [12].

When protein degradation surpasses synthesis, a decrease in muscle strength and muscle fiber CSA is observed [20]. Protein turnover is primarily regulated by the phosphatidylinositol-3-kinase (PI3K)/protein kinase B (Akt) signaling pathway, which when activated, protein synthesis is stimulated, while degradation is inhibited. Signaling is initiated when insulin, or insulin-like growth factor 1 (IGF-1), binds and activates the insulin receptor, which in turn phosphorylates insulin receptor substrate1, which ultimately activates the PI3K/Akt pathway [21]. Activated PI3K phosphorylates and activates Akt, localizing it to the plasma membrane. Subsequently, Akt activates the mammalian target of rapamycin (mTOR), which is present as mTOR complex 1 (mTORC1), and activated mTORC1 phosphorylates ribosomal protein S6 kinase 1 (S6K1) and eukaryotic translation initiation factor 4E(eIF4E)-binding protein 1 (4E-BP1) to stimulate protein synthesis. Also, Akt inhibits protein degradation via phosphorylation and location of forkhead box O3a (FoxO3a) from the nucleus to the cytosol [22]. Muscle protein degradation plays an essential role in skeletal muscle atrophy and muscle-wasting conditions through immobilization. FoxO regulates two muscle-specific E3 ligases, namely muscle ring finger 1 (MuRF1) and muscle atrophy F-box (Atrogin-1), both of which catalyze muscle protein degradation by degrading the ubiquitin-proteasome system [23].

The mitochondria serve various pivotal functions, such as energy production, regulation of intracellular calcium homeostasis, and integration of apoptosis signaling. Interfering with mitochondrial quality regulation can cause muscle atrophy. Therefore, mitochondrial function and content regulation are essential for maintaining muscle function and health [24]. PGC-1α, a transcription coactivator, induces mitochondrial biogenesis by stimulating transcription factors nuclear respirator factor1 and 2 (NRF-1 and NRF-2), which increase the transcription of mitochondrial enzymes [25]. The expression of the PGC-1α genes in fast-twitch muscles promotes mitochondrial biogenesis, oxidative metabolism associated with exercise, and resistance to muscle atrophy. This indicates that PGC-1α is essential for the prevention of sarcopenia. Also, NRF-1 and NRF-2 interact with TFAM to induce replication and transcription of the mtDNA [26].

Pacific oysters (*Crassostrea gigas*), one of the most widely distributed marine food resources, contain high-quality protein, taurine, minor elements, and zinc. Oyster proteins can be degraded into several peptides via enzymatic hydrolysis, and have prominent physiological activity. Furthermore, oyster hydrolysates have antioxidant, anti-inflammation [27], and anti-cancer [28,29] properties. However, the anti-muscle atrophy effect of oyster hydrolysates remains nebulous. Therefore, we prepared two different oyster hydrolysates: one was hydrolyzed with transglutaminase, protamex, and neutrase (TGPN); and the other was hydrolyzed with protamex, neutrase, and fermented by yeast (PNY). In this study, we investigate whether TGPN and PNY could prevent or delay muscle atrophy in dexamethasone-induced C2C12 cells and immobilization-induced mice by focusing on protein turnover and mitochondrial biogenesis-related pathways.

## 2. Materials and Methods

### 2.1. Preparation of TGPN and PNY

Pacific oysters (*Crassostrea gigas*) were collected from the nearby sea of Tongyeong, South Korea. The TGPN preparation process was performed as previously described [30,31]. Briefly, the oyster was hydrolyzed with 1% Protamex (Biosis, Busan, Korea) and 1% Neutrase (Biosis, Korea) for 2 h at 50 °C. Before hydrolysis with both proteases, they were pretreated with transglutaminase (Ajinomoto, Tokyo, Japan) for the crosslinking of oyster proteins.

The PNY preparation was conducted as previously reported [32]. The first step of PNY manufacturing was blanching at the boiled water for 5 min. Subsequently, three volumes of water were added to the mixture, which was homogenized with proteases Protamex (Biosis, Korea) and Neutrase (Biosis, Korea), and fermented by food-grade yeast (Societe Industrielle Lesaffre, Marcq-en-Baroeul, France). Proteases and yeast were inactivated by heating 30 min at 90 °C. PNY was filtered with 200 mesh screens, and the freeze-drying process was performed. TGPN and PNY were provided by Oceanpep Co. (Tongyeong, Korea).

### 2.2. Cell Culture

#### 2.2.1. Cell Culture

C2C12 myoblasts were purchased from American Type Culture Collection (ATCC No.CRL-1772™, Manassas, VA, USA).

C2C12 cells (mouse skeletal myoblasts) were cultured with growth media (DMEM) (ATCC, USA) supplemented with 10% fetal bovine serum (FBS; Gibco, Carlsbad, CA, USA) and 1% penicillin-streptomycin (Welgene, Gyeongsan-si, Korea) at 37 °C with 5% CO_2_. C2C12 myoblasts were planted until reaching 80% confluence; the growth media was replaced with differentiation media (DMEM supplemented with 2% horse serum (Gibco, USA), and 1% penicillin-streptomycin). After 7 days of differentiation, myoblasts were completely differentiated into myotubes. All the cell experiments were performed at least three times.

#### 2.2.2. Cell Viability Assay

Cell viability of TGPN and PNY was evaluated using a CCK-8 assay (Dogindo, USA). The C2C12 were seeded at 6 × 10^3^ cells per well on 96 well plates, and incubated at 37 °C, 5% CO_2_ until reaching 80% confluence. Cells were switched to differentiation media for up to 7 days, and treated with different concentrations of TGPN and PNY, and incubated in serum-free media for 48 h. Subsequently, CCK-8 reagent was added and incubated at 37 °C for an additional 1 h. The absorbance was measured at 450 nm using an ELISA microplate reader (Bio-Tek Instruments Inc., Winooski, VT, USA).

### 2.3. Treatment with Dexamethasone and TGPN, PNY

Following 7 days of differentiation, C2C12 myotubes were subdivided into 8 groups: a normal group, in which cells were incubated in serum-free medium (SFM; DMEM containing 1% penicillin); a control group, in which cells were treated with 50 μM dexamethasone (Sigma-Aldrich, St. Louis, MO, USA); Dex + TGPN groups, in which cells were treated with 50 μM dexamethasone and 100 μg/mL, 200 μg/mL, 400 μg/mL TGPN; Dex + PNY groups, in which cells were treated with 50 μM dexamethasone and 100 μg/mL, 200 μg/mL, 400 μg/mL PNY. All groups were incubated in SFM at 37 °C, 5% CO_2_ for 48 h before harvesting cells for experiments, which were performed at least three times.

### 2.4. Determination of C2C12 Myotube Diameter

The myotubes were washed with cold PBS three times, and fixed with 4% paraformaldehyde. Images of the C2C12 myotubes were captured using an optical microscope (Olympus, Tokyo, Japan). Ten myotube diameters were measured from each image, and the average value was used for quantification using Image J software (*n* = 10/well, six well were measured in each group).

### 2.5. Animals and Experimental Design

**Figure 1:**
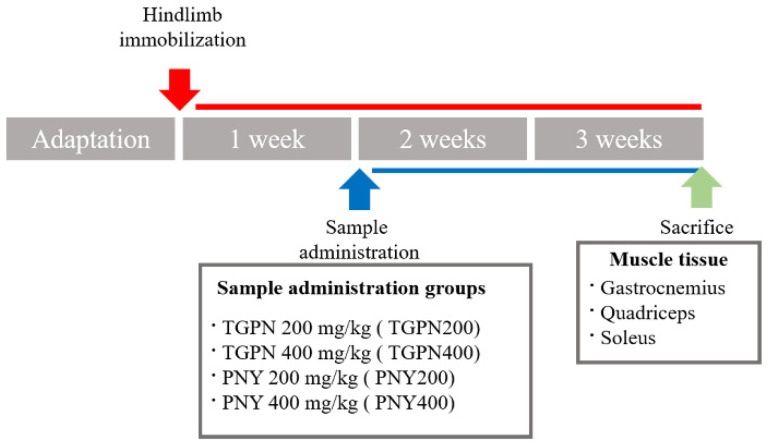
Animal experiment design.

Five-week-old male C57BL/6J mice were purchased from Raon Bio (Yongin, Korea), and housed in a controlled environment (25 ± 1 °C, 12 h:12 h light-dark cycle) with free access to food (Teklad Global 18% Protein Rodent Diet, ENVIGO, Indianapolis, IN, USA) and tap water. Mice were randomly divided into a normal group (*n* = 7) and an immobilization (IM) group (*n* = 35). Muscle atrophy was induced by hindlimb immobilization (Figure 1) [33]. After 1 week of IM, the IM group mice were randomly divided into five groups as follows (*n* = 7 per group): (I) immobilization-treated group (IM); (II) immobilization and 200 mg/kg/day TGPN-treated group (TGPN 200); (III) immobilization and 400 mg/kg/day TGPN-treated group (TGPN 400); (IV) immobilization and 200 mg/kg/day PNY-treated group (PNY 200); and (V) immobilization and 400 mg/kg/day PNY-treated group (PNY 400). The TGPN and PNY groups received an oral administration of TGPN and PNY for two weeks with continuous immobilization. Mice in the normal and IM groups were orally administered CMC (carboxymethyl cellulose). At the end of the administration period, treadmill running time and distance were measured. Then, the mice were sacrificed by cardiac puncture under anesthesia. The gastrocnemius, soleus, and quadriceps muscles were isolated and stored at −80 °C. The Institutional Animal Care approved the protocol of the animal study, and the committee guidelines of Kyung Hee University were used (approval number: KHSASP-20-228).

### 2.6. Muscle Function Test

#### 2.6.1. Grip Strength Test

The forelimb/hindlimb and forelimb grip strengths were measured with a grip strength meter (Bioseb, Chaville, France) every 3 days. Each mouse was allowed to hold the bar with their forelimb and hindlimb. The mouse’s tail was pulled back horizontally until the mouse released the grid. The maximum force of grip was measured. Five consecutive tests per mouse were performed for analysis. The grip strength was normalized to bodyweight [34].

#### 2.6.2. Treadmill Test

Treadmill running tests were performed using a treadmill (Columbus Instruments, Columbus, OH, USA). As an acclimation test, mice ran at 16 m/min for 10 min with no incline. For the actual test, mice ran at 12 m/min with no incline, and the speed was gradually increased by 3 m/min every 3 min until reaching 30 m/min. The test was terminated when mice could no longer run for 10 s.

### 2.7. H&E Staining

Isolated gastrocnemius muscles were fixed in 4% paraformaldehyde, embedded in paraffin, and sliced into 4 μm sections. Then, sections were stained with hematoxylin and eosin (H&E) staining solution for 13 h. Cross-sectional area (CSA) images were captured using an optical microscope. The CSA of myofibers was quantified using Image J software (64-bit Java 1.8.0_172).

### 2.8. Protein Extract and Western Blot Analysis

The C2C12 cells were rinsed three times with ice-cold PBS, and lysed using RIPA II Cell Lysis Buffer (1×) (GenDEPOT, Katy, TX, USA) containing a PhosSTOP EASY pack phosphatase inhibitor cocktail tablet (Roche, Mannheim, Germany) and protease inhibitor cocktails (Roche, Mannheim, Germany). Then, they were centrifuged at 13,000 rpm for 15 min at 4 °C. The supernatants were used for the western blot.

Gastrocnemius muscle tissue was homogenized with liquid nitrogen, and lysed using a lysis buffer containing a PhosSTOP™ and cOmplete™ protease inhibitor cocktail, then centrifuged at 13,000 rpm for 15 min at 4 °C, and the supernatants were used for the western blot. The protein concentrations were measured using the Pierce™ BCA protein assay kit (Thermo Fisher Scientific, Waltham, MA, USA) following the manufacturer’s instructions. Equal amount of protein from each group was loaded onto an 7.5%, 12%, or 15% polyacrylamide gel, and transferred onto a polyvinylidene fluoride (PVDF) membrane.

The membranes were blocked with 5% skim milk in the tris-buffered saline with Tween 20 (TBST) for 1 h at room temperature, and incubated overnight with the primary antibody diluted with 5% BSA in TBST at 4 °C

After washing with TBST for 30 min, a horseradish peroxidase-conjugated second antibody was added to the blocking solution (Santa Cruz Biotechnology Inc., Dallas, TX, USA), and incubated for 100 min.

The protein bands were detected using enhanced chemiluminescence and an image analyzer using an LAS3000 luminescent (Fuji Film, Tokyo, Japan), whereas protein expression levels were analyzed using Image J software, and normalized to the β-actin. The primary antibodies used were as follows, and diluted as 1:1000 with BSA: Cell Signaling (Danvers, MA, USA) (p-PI3K (#4228); Akt (#9272); p-Akt (#9271); p-mTOR (#2971); mTOR (#2972); p-S6K1 (#9205); S6K1 (#9202); p-4E-BP1 (#2855); 4E-BP1 (#9452); p-FoxO3a (#9465); FoxO3a (#12829) and SirT1 (#9475)); Abcam (Cambridge, UK) (PI3K (ab191606) and PGC-1α (ab54481)); GeneTex (CA, USA) (β-actin (GT5512)); and Santa Cruz Biotechnology (CA, USA) (Atrogin-1 (sc-166806) and MuRF1 (sc-398608)).

### 2.9. RNA Extract and Quantitative Real-Time Polymerase Chain Reaction (qRT-PCR) Analysis

Gastrocnemius muscle tissues were homogenized with liquid nitrogen, and total RNA was extracted using easy-RED™ (iNtRON, Seongnam, Korea) according to the manufacturer’s instructions. Total RNA was reverse-transcribed to cDNA using the PrimeScript™ 1st strand cDNA synthesis kit (TaKaRa, Tokyo, Japan) according to the manufacture’s instruction. After synthesizing cDNA, mRNA expression was analyzed by qRT-PCR using a Step One Plus™ Real-time PCR-System (Applied Biosystems, Foster City, CA, USA) with TB Green™ Premix Ex Taq™ (TaKaRa, Tokyo, Japan). The data were normalized to *GAPDH*, and calculated using a comparative method (2^−ΔΔCt^). Table 1 lists the primer sequences used in this study.

### 2.10. Quantitative Analysis of Mitochondrial DNA

A total of 25–50 mg of gastrocnemius muscle tissues were homogenized with liquid nitrogen, and total DNA was extracted using an AccuPrep^®^ Genomic DNA Extraction Kit (Bioneer, Daejeon, South Korea) according to the manufacturer’s instructions. To qualify the mtDNA content, qRT- PCR was performed as described above. The data were quantified by taking the ratio between mtDNA (mitochondrial DNA)/nDNA (nuclear DNA). Table 2 lists the primer sequences.

### 2.11. Statistical Analysis

Data were presented as the mean ± standard deviation (SD). The statistical significance of differences between groups was determined using one-way ANOVA of Tukey’s post hoc test (SPSS 25, Chicago, IL, USA). Statistical significance was shown as follows: # *p* < 0.05; ## *p* < 0.01 and ### *p* < 0.001 compared to the normal group; and * *p* < 0.05, ** *p* < 0.01, and *** *p* < 0.001 compared to the Dex or IM group.

## 3. Results

### 3.1. TGPN and PNY Protected against Dexamethasone-Induced Muscle Atrophy in C2C12

**Figure 2:**
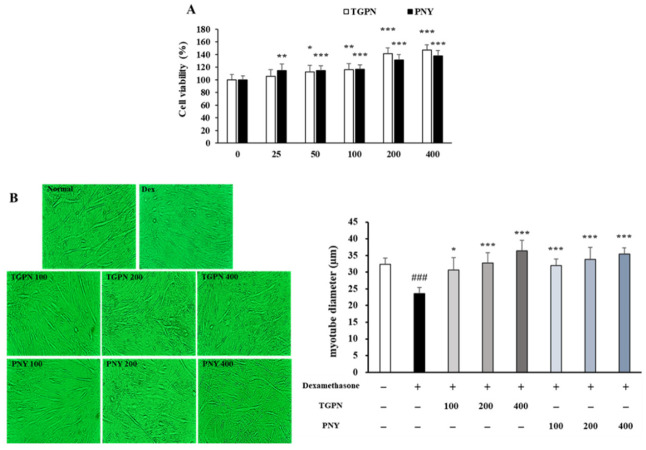
Effect of TGPN and PNY on cell viability and myotube atrophy in C2C12 myotubes. (**A**) Cell viability of TGPN and PNY. C2C12 myotubes were treated with various concentrations (100, 200, 400 μg/mL) of TGPN and PNY on day 7 of differentiation for 48 h. (**B**) Quantification of C2C12 myotube diameter and 400x magnification images of myotubes. 50 μM dexamethasone and TGPN or PNY (100, 200, 400 μg/mL) were co-treated on day 7 of differentiation for 48 h. The data are expressed as mean ± SD. ### *p* < 0.001 versus normal, * *p* < 0.05, ** *p* < 0.01, *** *p* < 0.001 versus untreated control or Dex.

Prior to assessing the protective effects of TGPN and PNY on myotube atrophy, the CCK-8 assay was used to determine the potential toxicity of TGPN and PNY to C2C12 myotubes. Cell viability was measured with diverse concentrations (25, 50, 100, 200, and 400 μg/mL) of TGPN or PNY in the C2C12 myotube. The cell viability of TGPN and PNY was increased in a concentration-dependent manner until 400 μg/mL without toxicity, suggesting that TGPN and PNY treatment significantly promoted C2C12 cell proliferation (Figure 2A). Therefore, we used 100, 200, and 400 µg/mL as concentrations for TGPN and PNY.

As shown in Figure 2B, the C2C12 myotube diameter reduced by 27% in the Dex group, indicating dexamethasone-induced atrophy in C2C12 myotubes. Conversely, the myotube diameter of the TGPN and PNY treatment groups increased in a concentration-dependent manner, and at 200 and 400 μg/mL, the TGPN and PNY restored the myotube diameter to the normal level.

**Figure 3:**
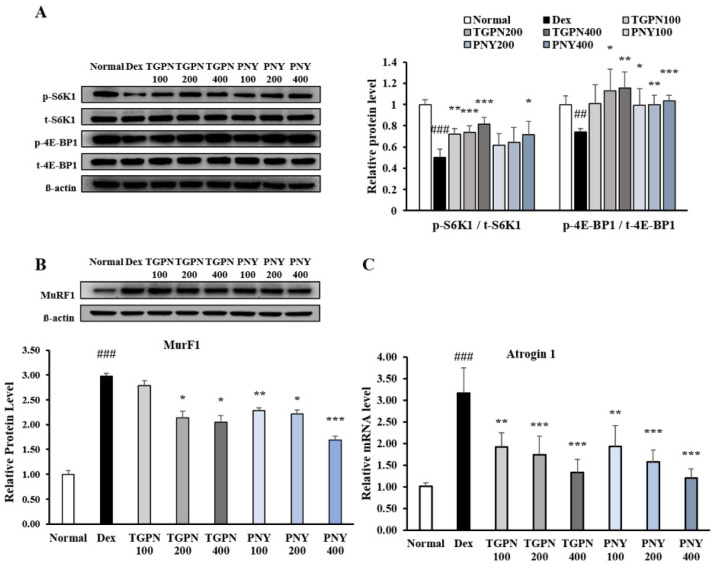
Effect of TGPN and PNY on expression associated with protein synthesis and degradation in C2C12 myotubes. C2C12 myotubes were co-treated with 50 μM dexamethasone and various concentrations (100, 200, 400 μg/mL) of TGPN and PNY on day 7 of differentiation for 48 h. The relative expression levels of (**A**) S6K1 and 4E-BP1, (**B**) MuRF1, (**C**) Atrogin-1. The protein expression levels were normalized to the β-actin. The mRNA expression level was normalized to GAPDH. The results are presented as mean ± SD. ## *p* < 0.01, ### *p* < 0.001 versus normal, * *p* < 0.05, ** *p* < 0.01, *** *p* < 0.001 versus Dex.

We next investigated the effect of TGPN and PNY on molecular mechanisms involved in the PI3K/Akt pathway regulating protein turnover. The phosphorylation ratio of S6K1 and 4E-BP1, the sub-factors of protein synthesis in the PI3K/Akt pathway, showed a significant increase in a concentration-dependent manner (Figure 3A). Furthermore, MuRF1 protein expression was significantly decreased compared to the Dex group, with Atrogin-1 mRNA expression showing similar patterns (Figure 3B). Interestingly, TGPN was more effective in activating the markers associated with protein synthesis, whereas PNY was more effective in inhibiting the markers associated with protein degradation. These results suggest that TGPN and PNY have an anti-muscle atrophic effect through the PI3K/Akt signaling pathway.

### 3.2. TGPN and PNY Ameliorated Muscle Function in Immobilization-Induced Muscle Atrophy Mice

**Figure 4:**
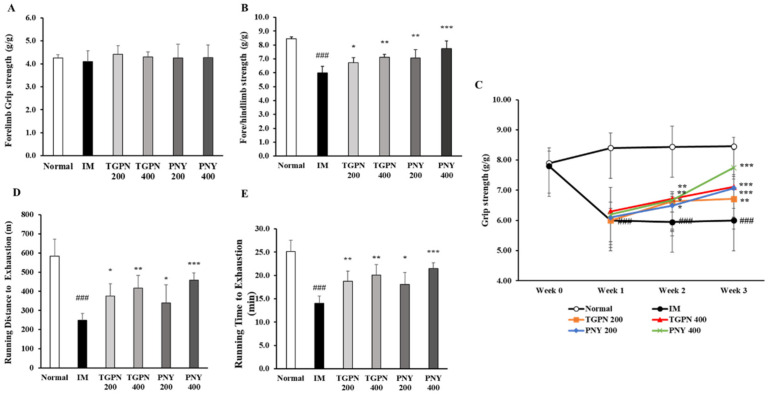
Effect of TGPN and PNY on muscle function in hindlimb immobilization-induced muscle atrophy mice. Mice were subjected to hindlimb immobilization for one week, except for the normal group, and then administered with TGPN 200, 400 mg/kg, or PNY 200, 400 mg/kg for two weeks with continuous immobilization. (**A**) The forelimbs grip strength. (**B**) The fore/hindlimbs grip strength. (**C**) The grip strengths curve. The grip strengths were normalized to the bodyweight. (**D**) Running distance on the treadmill. (**E**) Running time on the treadmill. Data are presented as the mean ± SD. ### *p* < 0.001 versus normal, * *p* < 0.05, ** *p* < 0.01, *** *p* < 0.001 versus IM.

Loss of muscle function and strength is one of the main characters of sarcopenia [35]. We assessed the effect of TGPN and PNY on muscle function by evaluating grip strength and exercise endurance. Interestingly, when measuring the grip strength to evaluate muscle exercise capacity, we observed no significant differences between the groups in the forelimb grip strength (Figure 4A). However, the fore/hindlimb grip strength showed a completely different trend from the forelimb strength (Figure 4B). The fore/hindlimb grip strength of the IM group continued to decrease during the experimental period, whereas the administration group significantly increased by 29%, 19%, 18%, and 12% in the order of PNY 400 ≫ TGPN 400 ≒ PNY 200 > TGPN 200 compared to the IM group (Figure 4C).

In the treadmill test used to evaluate exercise endurance, the running time and distance to exhaustion were significantly decreased in the IM group compared to the normal group. Furthermore, those parameters in the TGPN and PNY groups were significantly increased dose-dependently. Among the administration groups, the PNY 400 was the most effective for exercise endurance (Figure 4D,E). To sum up, the TGPN and PNY significantly increased both grip strength and exercise endurance, with PNY being superior to TGPN in improving muscle function.

### 3.3. TGPN and PNY Increased Muscle Mass and Cross-Sectional Area of Muscle Fiber in Immobilization-Induced Muscle Atrophy Mice

The muscle mass and CSA of muscle fibers have been reported to decrease in immobilization-induced muscle atrophy [36]. Therefore, we determined whether TGPN and PNY administration could affect the muscle mass and CSA of muscle fibers. After sacrifice, we collected three different skeletal muscle tissues: gastrocnemius; quadriceps; and soleus; and evaluated the muscle mass by normalizing to the bodyweight.

**Figure 5:**
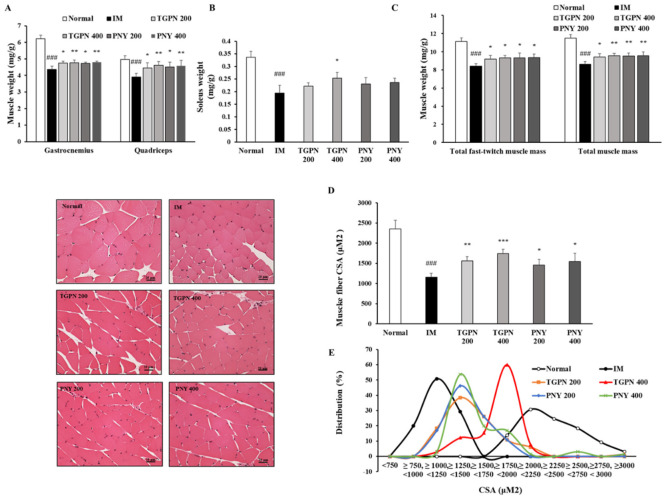
Effect of TGPN and PNY on body composition. Mice were subjected to hindlimb immobilization for one week, except for the normal group, and then administered with TGPN 200, 400 mg/kg, or PNY 200, 400 mg/kg for two weeks with continuous immobilization. (**A**) The fast-twitch muscle mass (gastrocnemius, quadriceps). (**B**) The slow-twitch muscle mass (soleus). (**C**) The total fast-twitch muscle mass and total muscle mass. Muscle mass was normalized to the bodyweight. (**D**) The cross-sectional area (CSA) of gastrocnemius muscle fiber. (**E**) The distribution graph of muscle fiber CSA. The scale bar is 25 μm. The data are shown as mean ± SD. ### *p* < 0.001 versus normal, * *p* < 0.05, ** *p* < 0.01, *** *p* < 0.001 versus IM.

In terms of muscle mass, the fast-twitch muscles (gastrocnemius and quadriceps) were significantly decreased by 25% and 22%, and the slow-twitch muscle (soleus) was significantly reduced by 42% in the IM group compared to the normal group. Consistent with previous findings, immobilization altered the characteristic properties of slow-twitch fibers in comparison with the fast-twitch fibers [37], i.e., the slow-twitch muscle was further reduced compared with the fast-twitch muscle. The TGPN administration groups significantly increased in all three muscle masses. Meanwhile, the PNY administration groups significantly increased the fast-twitch muscle mass, whereas the slow-twitch muscle mass showed a tendency to increase (Figure 5A,B). In the total muscle mass, both TGPN and PNY administration groups significantly alleviated in a dose-dependent manner by 11%, 11%, 10%, and 10% in the order of PNY 400 = TGPN 400 > PNY 200 > TGPN 200 compared to the IM group (Figure 5C).

In addition, the CSA of the gastrocnemius muscle was also consistently enlarged, suggesting that TGPN and PNY affect the size of muscle fibers. When comparing the CSA in the TGPN and PNY administration groups, the TGPN administration groups showed the most significant increase (Figure 5D). In the CSA distribution graph, the muscle fiber of the IM group leaned to the left, and the muscle fibers were distributed between 750 mM^2^ and 1500 mM^2^. The muscle fibers of the normal group were evenly distributed on the right side, and are concentrated between 1750 mM^2^ and 3000 mM^2^. The TGPN 200, and PNY 200 and 400 administration groups were extensively distributed between 1240 mM^2^ and 1750 mM^2^. TGPN 400 group peaked between 1750 mM^2^ and 2250 mM^2^, whereas the chart was the most similar to the normal group (Figure 5E).

### 3.4. TGPN and PNY Stimulated Muscle Protein Synthesis and Blocked Muscle Protein Degradation via PI3K/Akt Pathway in Immobilization-Induced Muscle Atrophy Mice

**Figure 6:**
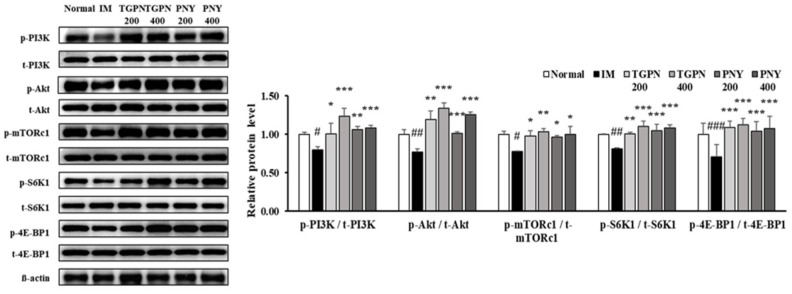
Effect of TGPN and PNY on muscle protein synthesis mechanism in gastrocnemius muscle. Mice were subjected to hindlimb immobilization for one week, except for the normal group, and then administered with TGPN 200, 400mg/kg, or PNY 200, 400 mg/kg for two weeks with continuous immobilization. Relative phosphorylation of PI3K, Akt, mTORc1, S6K1, and 4E-BP1. The protein expression levels were normalized to the β-actin level. The data are expressed as mean ± SD. # *p* < 0.05, ## *p* < 0.01, ### *p* < 0.001 versus normal, * *p* < 0.05, ** *p* < 0.01, *** *p* < 0.001 versus IM.

The gastrocnemius is the largest part of the three muscles that make up the hindlimb, and the gastrocnemius muscle has a high capacity for both force and power production. Therefore, we studied the molecular mechanism using the gastrocnemius muscle. Activation of the PI3K/Akt pathway increases protein turnover through simultaneous regulation of protein anabolism and catabolism. First, the PI3K/Akt/mTORc1 pathway regulates muscle protein synthesis [38]. The phosphorylation ratio of PI3K and Akt was significantly decreased during IM, which was followed by a significantly reduced phosphorylation ratio of mTOR, S6K1, and 4E-BP1. Administration of TGPN and PNY dose-dependently alleviated these parameters, and restored normal levels. In terms of protein synthesis, the TGPN groups showed superior results to that of the PNY groups, consistent with our in vitro results (Figure 6).

**Figure 7:**
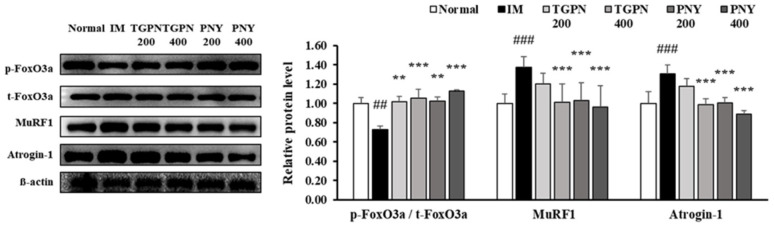
Effect of TGPN and PNY on muscle protein degradation mechanism in gastrocnemius muscle. Mice were subjected to hindlimb immobilization for one week, except for the normal group, and then administered with TGPN 200, 400 mg/kg, or PNY 200, 400 mg/kg for two weeks with continuous immobilization. Relative phosphorylation of FoxO3a, and relative protein levels of MuRF1 and Atrogin-1. The protein expression levels were normalized to the β-actin level. The data are expressed as mean ± SD. ## *p* < 0.01, ### *p* < 0.001 versus normal, ** *p* < 0.01, *** *p* < 0.001 versus IM.

The PI3K/Akt/FoxO3a pathway regulates the ubiquitin protease muscle protein degradation pathway. When the FoxO3a pathway is activated, the phosphorylation of FoxO3a prevents the translocation of FoxO3a from the cytoplasm into the nucleus [39]. Therefore, p-FoxO3a downregulates MuRF1 and Atrogin-1, which are E3 ligase. The p-FoxO3a/t- FoxO3a ratio significantly decreased in the IM group, whereas this ratio increased to the normal level in all TGPN and PNY groups. Consequently, MuRF1 and Atrogin-1 protein expression levels were significantly increased in the IM group, and decreased by TGPN and PNY administration in a dose-dependent manner. In terms of protein degradation, the PNY groups were more effective compared to the TGPN groups (Figure 7). Taken together, TGPN and PNY administration attenuated the imbalance of protein turnover in a dose-dependent manner, and recovered to the normal level in the TGPN 400 and PNY 400 groups.

### 3.5. TGPN and PNY Improved Mitochondrial Biogenesis in Immobilization-Induced Muscle Atrophy Mice

**Figure 8:**
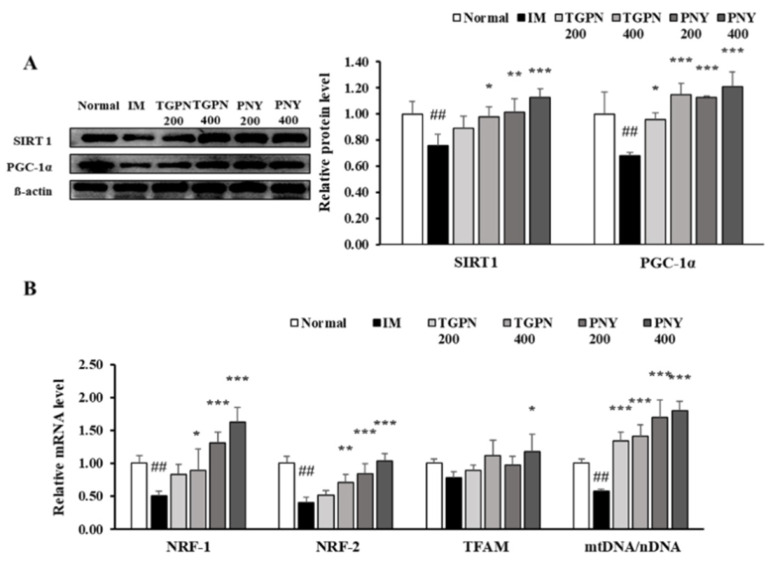
Effect of TGPN and PNY on mitochondrial biogenesis mechanism in gastrocnemius muscle. Mice were subjected to hindlimb immobilization for one week, except for the normal group, and then administered with TGPN 200, 400 mg/kg, or PNY 200, 400 mg/kg for two weeks with continuous immobilization. (**A**) Relative protein levels of SIRT1 and PGC-1α. They were normalized to the β-actin level. (**B**) Relative mRNA levels of *NRF-1, NRF-2 TFAM*, and mtDNA contents. The mtDNA content quantified by taking the ratio between mtDNA/nDNA. The mRNA expression level was normalized to GAPDH. The data are expressed as mean ± SD. ## *p* < 0.01 versus normal, * *p* < 0.05, ** *p* < 0.01, *** *p* < 0.001 versus IM.

In this study, TGPN and PNY affected the grip strength and muscular endurance test, hence, we determined whether any association with mitochondrial biogenesis exists. NAD-dependent deacetylase sirtuin-1 (SIRT1) activates PGC-1α by deacetylation of lysine residues, and PGC-1α regulates transcription factor NRF-1/2 and TFAM. Consequently, activation of SIRT1/PGC-1α signaling enhances mitochondrial biogenesis. In the IM group, the protein expression of SIRT1 and PGC-1α levels were significantly decreased compared to those in the normal group. TGPN and PNY administration significantly increased the downregulated protein expression of SIRT1 and PGC-1α; notably, the expression level of both markers in the PNY 400 group was restored to normal level (Figure 8A). Subsequently, the mRNA expression of *TFAM, NRF-1, and NRF-2*, which related to mitochondrial biogenesis, were decreased in the IM group compared to the normal group. These decreases were alleviated in the TGPN and PNY groups. Consistently, relative mtDNA content was significantly decreased in the IM group versus the normal group. TGPN and PNY administration, however, dose-dependently increased mtDNA content (Figure 8B). Overall, these results suggest that TGPN and PNY simulate mitochondrial metabolism and SIRT1/PGC-1α signaling.

## 4. Discussion

The present study aimed to verify the inhibitory effect and metabolic changes of oyster hydrolysates on muscle atrophy using C2C12 cell lines and mice. We hydrolyzed oysters into two different oyster protein hydrolysates, TGPN and PNY. First, we confirmed the anti-sarcopenic effects of TGPN and PNY on dexamethasone-induced atrophy in C2C12 myotubes. C2C12 cells are regularly used to study signaling pathways related to skeletal muscle [40]. As myoblasts differentiate into myotubes, the number of myofibers increases due to fusion between cells, and the diameter of myofibers thickens. However, when muscle atrophy is induced, the number and diameter of muscle fibers decreases through the breakdown of muscle protein, thereby decreasing overall muscle mass. Therefore, to induce such muscle atrophy, 50 μM dexamethasone was used as a muscle atrophy inducer [41,42]. In the Dex group, mature myotube diameter was significantly decreased compared with the normal group, suggesting that 50 μM dexamethasone was an appropriate concentration to induce myotube atrophy. However, the myotube diameter was restored to normal levels following treatment with TGPN and PNY at concentrations of 200 and 400 μg/mL, respectively. Therefore, our in vitro results suggest that TGPN and PNY have protective effects on muscle atrophy.

Various models of sarcopenia have been previously used, such as denervation, cast immobilization, and hindlimb unloading [38]. Previous studies have shown that hindlimb-immobilization significantly reduced muscle mass, exercise endurance, grip strength, and muscle fiber CSA [43,44,45]. Our study showed similar changes, and, thus, we verified the pathogenesis of sarcopenia by hindlimb immobilization. At the end of the experiment, the bodyweight of the IM group was reduced by approximately 6% compared with the normal group, indicating that muscle wasting may have been caused by immobilization (Supplementary Figure S1). However, the bodyweight of TGPN and PNY administration groups showed a tendency to increase compared to the IM group, and both the 400 mg/kg TGPN and PNY administration groups showed increased bodyweight, similar to that of the normal group. In terms of total muscle mass, the TGPN 400 and PNY 400 administration groups showed a significant increase of 11% compared to the IM group, and the increased muscle mass may be contributed to the increase of bodyweight. This result can be proof that muscle loss by immobilization was recovered by TGPN and PNY administration.

Skeletal muscle fibers provide the energy needed for physical activity. The fast-twitch fiber uses glycolytic metabolism, and is required for short duration and high intensity physical activities, such as sprinting and strength training. This mainly relies on type II muscle fibers, such as the gastrocnemius and quadriceps; whereas the slow-twitch fiber primarily uses oxidative metabolism, and is needed for long duration and low intensity physical activities, such as distance running and endurance training. This is primarily dependent on type I muscle fibers, such as the soleus. Persistent muscle inactivity causes slow-twitch fiber atrophy, and age-induced atrophy causes fast-twitch fiber loss. Muscle endurance is an important part of physical performance, and is used to evaluate musculoskeletal function [46]. In our study, running distance and time to exhaustion were significantly decreased in the IM group, and were recovered in the TGPN and PNY administration groups. These parameters were associated with the slow-twitch muscle soleus, which showed a tendency to increase in the TGPN and PNY administration groups compared to the IM group.

We then attempted to elucidate the molecular mechanisms underlying the anti-muscle atrophy effect of TGPN and PNY. Muscle atrophy occurs due to an imbalance in protein synthesis and degradation. Therefore, we investigated the PI3K/Akt pathway that mainly regulates protein turnover [47]. The PI3K/Akt/mTORc1 pathway increases protein synthesis by regulating downstream factors S6K1 and 4E-BP1. TGPN and PNY administration stimulated PI3K and Akt protein expression by phosphorylating mTORc1, S6K1, and 4E-BP1, leading to enhanced muscle protein synthesis; whereas the PI3K/Akt/FoxO3a pathway decreases protein degradation by regulating downstream factors MuRF1 and Atrogin-1. FoxO3a is a transcription factor of E3 ubiquitin ligases Atrogin-1 and MuRF1. Two E3 ubiquitin ligases promote protein degradation via the ubiquitin-proteasome system. TGPN and PNY administration enhanced the phosphorylated FoxO3a protein expression, and downregulated Atrogin-1 and MuRF1 protein expression, leading to inhibition of muscle protein degradation. To sum up, TGPN and PNY administration restored the imbalance of protein turnover by improving the PI3K/Akt pathway. In vitro, TGPN significantly increased the expression of 4E-BP1 and S6K1, the lowest factors for muscle protein synthesis. In contrast, PNY inhibited muscle protein degradation by significantly decreasing the muscle-specific ubiquitin E3 ligases MuRF1 and Atrogin-1. These tendencies were also the same in vivo. TGPN administration groups showed a more significant increase in all muscle protein synthesis-related factors, whereas PNY administration groups were more inhibited in all muscle protein degradation-related factors.

Mitochondria are the most prominent energy source in skeletal muscle [40]; hence, mitochondrial quality is a new strategy for preventing muscle atrophy [41,42]. Improvement of mitochondrial biogenesis can be accomplished with not only muscle strength, but also endurance performance. Therefore, we used the grip strength to test the muscle strength. Grip strength tests have been used to predict overall body strength and decline in muscle function [46]. We measured the grip strength weekly, and observed a significant increase from the first week of administration. After 2 weeks of administration, the grip strength gap between the administration groups was further widened. Compared to the IM group, the grip strength showed a significant increase in the order of PNY 400 > PNY 200 = TGPN 400 > TGPN 200. Also, due to muscle fiber CSA using fast-twitch muscles, a significant increase was observed in the order of TGPN 400 > TGPN 200 = PNY 400 > PNY 200. Interestingly, though TGPN had a better effect on muscle fiber CSA than PNY, the PNY showed an improved effect on muscle function compared to TGPN, and no difference was observed in the increase of total muscle mass between the TGPN or PNY administration groups. To sum up, the administration of TGPN and PNY showed significant alleviation of both persistent muscle inactivity and age-induced muscle atrophy.

We also investigated the molecular mechanism underlaying muscle strength in terms of mitochondrial biogenesis. SIRT1 is a member of the sirtuin family of protein, and stimulates PGC-1α. PGC-1α is a key factor in regulating energy metabolism in muscle, and it increases the energy level by promoting mitochondrial biogenesis. In addition, PGC-1α activates the transcription factors affecting mitochondrial proliferation, energy homeostasis regulation, and respiration, such as NRF-1, NRF-2, and TFAM. In this study, to confirm the energy-enhancing effect of TGPN and PNY in muscle tissue, the expression changes of *NRF-1, NRF-2*, and *TFAM* genes were assessed, which showed that the expression of these regulators were upregulated by TGPN and PNY administration, which increased the mtDNA contents. The TGPN and PNY administration group recovered to the normal level. These results suggest that TGPN and PNY promote mitochondrial biogenesis by regulating the expression of energy metabolism regulators, possibly associated with an increase in slow-twitch muscle mass, and exercise endurance time and distance.

In this study, we identified the effect and molecular mechanisms of TGPN and PNY in C2C12 cells and immobilization-induced muscle atrophy mice. TGPN and PNY are peptides, and we thought that oyster hydrolysates have the potential to affect both protein turnover and mitochondrial biogenesis pathways. First, one of the peptides can have an effect by directly binding to the insulin-like growth factor 1 (IGF-1) receptor or targeting the PI3K/Akt signaling pathway. Second, oyster hydrolysates may stimulate the secretion of hormones, such as testosterone, estrogen, and growth hormone (GH). Therefore, we are now conducting advanced research to confirm this.

## 5. Conclusions

Two types of oyster hydrolysates (TGPN and PNY) enhanced C2C12 myotube diameter reduced by dexamethasone treatment. Also, TGPN and PNY significantly increased muscle function-related factors: grip strength; exercise endurance; muscle mass; and muscle fiber CSA. These results are possibly attributed to the recovery of protein synthesis and degradation balance, and improved mitochondrial biogenesis. TGPN and PNY activated not only muscle protein turnover (PI3K/Akt pathway), but also mitochondrial biogenesis (SIRT1/PGC-1α pathway). TGPN had a better effect on promoting CSA of muscle fibers and muscle protein synthesis, whereas PNY had an improved effect on muscle function, inhibited muscle protein degradation, and stimulated mitochondrial biogenesis. Collectively, TGPN and PNY could be used as an effective natural supplement for muscle atrophy through enhancing protein turnover and mitochondrial biogenesis.

## Figures and Tables

**Table 1:** List of primer sequences used for qRT-PCR.

Gene	Forward (5′-3′)	Reverse (5′-3′)
*Atrogin-1*	AGAAAGAAAGACATTCAGAACA	GCTCCTTCGTACTTCCTT
*TFAM*	CACCCAGATGCAAAACTTTCAG	CTGCTCTTTATACTTGCTCACAG
*NRF-1*	CGGTAGCATCACTGGCAGAA	GGATCTGGACCAGGCCATTA
*NRF-2*	TGAAGTTCGCATTTTGATGGC	CTTTGGTCCTGGCATCTCTAC
*GAPDH*	CGGCCGCATCTTCTTGTG	CCGACCTTCACCATTTTGTCTAC

**Table 2:** List of primer sequences used in qRT-PCR for mtDNA content.

Gene	Forward (5′-3′)	Reverse (5′-3′)
mtDNA	CCTATCCTTGCCATCAT	GAGGCTGTTGCTGTGAC
nDNA	ATGGAAAGCCTGCCATCATG	TCCTTGTTGTTCAGCATCAC

## Data Availability

Data is contained within the article and the Supplementary Materials.

## Supplementary Materials

The following are available online at https://www.mdpi.com/article/10.3390/nu13124385/s1, Figure S1: Bodyweight.
