# Supplementary material for: Oyster Hydrolysates Attenuate Muscle Atrophy via Regulating Protein Turnover and Mitochondria Biogenesis in C2C12 Cell and Immobilized Mice
Source: Nutrients. 2021 Dec 8;13(12):4385. doi: 10.3390/nu13124385 (PMC8703783; doi:10.3390/nu13124385)
Supplement: Supplementary file 1 [file nutrients-13-04385-s001.zip › nutrients-1468510-SI.pdf]

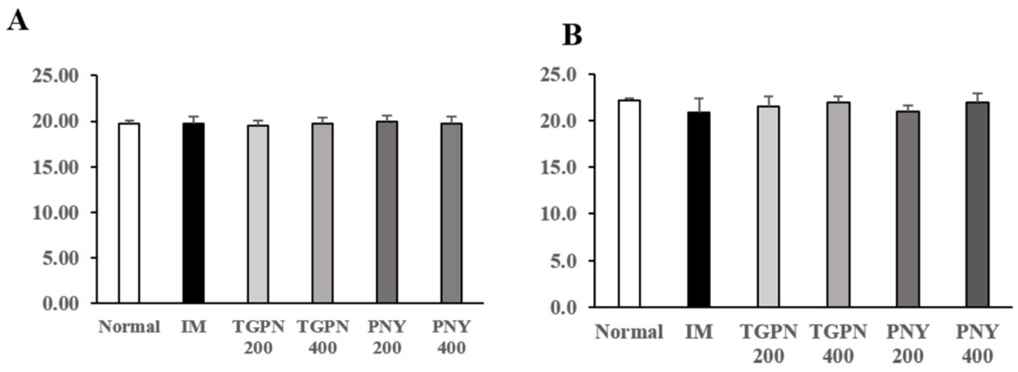

Supplementary Figure S1. Body weight.

(A) Body weight before the start of the administration. (B) Body weight at the end of the experiment.
